# rSK1 in Rat Neurons: A Controller of Membrane rSK2?

**DOI:** 10.3389/fncir.2019.00021

**Published:** 2019-04-03

**Authors:** Eleonora Autuori, Petra Sedlak, Li Xu, Margreet C. Ridder, Angelo Tedoldi, Pankaj Sah

**Affiliations:** Queensland Brain Institute, The University of Queensland, Brisbane, QLD, Australia

**Keywords:** spike frequency adaptation, potassium channel, afterhyperpolarization, excitability, calcium activated K^+^ channels (KCa1–KCa5)

## Abstract

In mammalian neurons, small conductance calcium-activated potassium channels (SK channels) are activated by calcium influx and contribute to the afterhyperpolarization (AHP) that follows action potentials. Three types of SK channel, SK1, SK2 and SK3 are recognized and encoded by separate genes that are widely expressed in overlapping distributions in the mammalian brain. Expression of the rat genes, rSK2 and rSK3 generates functional ion channels that traffic to the membrane as homomeric and heteromeric complexes. However, rSK1 is not trafficked to the plasma membrane, appears not to form functional channels, and the role of rSK1 in neurons is not clear. Here, we show that rSK1 co-assembles with rSK2. rSK1 is not trafficked to the membrane but is retained in a cytoplasmic compartment. When rSK2 is present, heteromeric rSK1-rSK2 channels are also retained in the cytosolic compartment, reducing the total SK channel content on the plasma membrane. Thus, rSK1 appears to act as chaperone for rSK2 channels and expression of rSK1 may control the level of functional SK current in rat neurons.

## Introduction

Potassium channels are widely expressed in the central nervous system (CNS) where they play an important role in regulating the intrinsic excitability of neurons. A subset of potassium channels expressed in central neurons are Ca^2+^-activated K^+^ channels that regulate cellular excitability and spike frequency adaptation (Coetzee et al., 1999; Adelman et al., 2012). These are divided into three families that comprise the large conductance (BK) channels (KCa1.1), small conductance (SK) channels KCa2.1, KCa2.2, KCa2.3 (SK1, SK2, and SK3), and the intermediate conductance (IK) channels KCa3.1 (Vergara et al., 1998). In neurons, these channels are generally driven by calcium influx during action potentials, and activation of BK currents contributes to spike repolarization, while SK channel activity is slower, contributing to the afterhyperpolarization (AHP) that follows (Sah, 1996). These channels can also be activated by calcium release from intracellular stores again leading to inhibition of neural activity (Fiorillo and Williams, 1998), and are also expressed at glutamatergic synapses, where they are activated by calcium influx during synaptic activity and play a role in tuning synaptic plasticity (Faber et al., 2005; Ngo-Anh et al., 2005; Lin et al., 2008).

SK channels are encoded by three genes, SK1, SK2 and SK3 (Köhler et al., 1996; Stocker and Pedarzani, 2000; Sailer et al., 2002), which are expressed throughout the mammalian brain in distinct but partially overlapping distributions. In the rodent brain, SK1 and SK2 are generally co-expressed while SK3 channels are present in a complementary distribution (Stocker and Pedarzani, 2000). Functional studies in heterologous expression systems have shown that rat SK2 (rSK2) and SK3 (rSK3), and human SK1 (hSK1) channels form functional homomeric channels that are voltage-insensitive and gated by the binding of Ca^2+^ to calmodulin (CaM; Xia et al., 1998; Lee and MacKinnon, 2018), which is covalently linked at their cytosolic, carboxy-terminal region (Lee and MacKinnon, 2018). In contrast, the rat SK1 (rSK1), does not generate functional homomeric channels (Köhler et al., 1996; D’Hoedt et al., 2004), but does appear to co-assemble with rSK2 (Ishii et al., 1997; Benton et al., 2003; Church et al., 2015). This difference is due to differences in the sequence identity of the two channels. Thus, rSK2 and rSK3 are highly homologous to the human SK2 (97.6%) and SK3 (94.4%), and have similar pharmacology and functional profiles (Köhler et al., 1996; Joiner et al., 1997; Desai et al., 2000), while rSK1 is only 84% homologous to hSK1 (D’Hoedt et al., 2004). Indeed, replacing the carboxy terminal of rSK1 with that of rSK2 restores functional expression of rSK1, and swapping C- and N-termini of hSK1 with those from rSK1 prevents expression of functional hSK1 channels on the cell membrane of HEK293T cells (D’Hoedt et al., 2004).

In summary, while rodent rSK1, rSK2 and rSK3 channels are widely expressed in neurons of the rodent nervous system, rSK1, unlike rSK2 and rSK3, does not form functional homomeric channels. Homomers and dimers consisting of SK2 and SK3 form functional channels and contribute to the AHP and are also involved in synaptic plasticity (Adelman et al., 2012). It has been reported (Benton et al., 2003) that co-expression of rSK1 and rSK2 in HEK293 cells results in larger calcium-activated currents with altered pharmacology suggesting that rSK1 can form functional heteromeric assemblies with rSK2, though biochemical evidence for this co-assembly is lacking. Thus, while rSK1 is widely co-expressed with rSK2, the functional role of rodent SK1 remains unclear. In this study, we test the function of rSK1 channels in a heterologous system and cultured rat hippocampal pyramidal neurons. Firstly, we tested if rSK1 and rSK2 could interact in a heterologous system. We then looked at the effect of either overexpressing or downregulating rSK1 channels and tested the level of rSK2 channels expressed on the cells membranes of both a heterologous system and in hippocampal pyramidal neurons. Finally, we tested if changing rSK1 levels was related to a change in medium AHP (mAHP) and its underlying current (I_AHP_) in hippocampal pyramidal neurons that are generated by the activation of SK2 channels (Stocker et al., 1999, 2004).

## Materials and Methods

### Neuronal Cultures

Primary E18 Wistar rat hippocampal cultures were prepared as previously described (Delaney et al., 2013). Briefly, hippocampi were digested for 20 min at 37°C in papain (12 U/ml, Worthington, suspension 28.4 U/mg) made up in dissection medium (1× HBSS, 1% penicillin/streptomycin, 1% pyruvate, 10 mM HEPES, 30 mM glucose) and supplemented with 1% DNase (Sigma). The digested material was washed three times in plating medium [Neurobasal medium (Life Technologies) supplemented with 5% heat-inactivated fetal bovine serum (FBS), 1% penicillin/streptomycin, 1% Glutamax (Life Technologies) and 2% B27 (Life Technologies)] and the resulting pellet was triturated in plating medium using a fire-polished glass Pasteur pipette. Cells were plated at a density of 1 × 10^5^ cells/ml for immunocytochemistry and the biotinylation assays and at 4 × 10^4^ cells/ml for the electrophysiology experiments, on glass coverslips precoated with poly-D-lysine in plating medium and maintained at 37°C in 5% CO_2_.

### Biotinylation and Western Blot Analysis

Hippocampal neurons were cooled on ice and were then rinsed three times with phosphate buffered saline (PBS) prior to exposure with EZ-Link Sulfo-NHS-SS-Biotin (Pierce), dissolved in PBS to a concentration of 1.22 mg/ml. The biotinylation reaction was undertaken on ice for 30 min, followed by biotin removal and washing twice with 100 mM glycine in PBS and then once with PBS. Cells were lysed in 200 μl lysis buffer (20 mM sodium phosphate, pH 7.5, 150 mM NaCl, 0.5% NP40, 0.5% sodium deoxycholate, 0.1% SDS, 1× complete protease inhibitors, Roche) and left on ice for 30–45 min. The lysates were then centrifuged at 6,500 *g* for 5 min. Twenty microliters of the resulting supernatant was then removed and this was classified as the total lysate (LYS). Biotinylated proteins were captured from the supernatant by the addition of 25 μl Streptavidin Agarose Resin (Pierce) at 4°C for 30 min. The beads were then centrifuged at 10,000 *g* for 1 min and the resulting supernatant was classified as the cytoplasmic phase (CYTO). The beads were washed a further three times in PBS. This was classed as the membrane fraction (MEMB). All samples were then boiled for 5–10 min in 1× SDS sample buffer containing 100 mM DTT and then fractionated on 4%–12% Bis-Tris gradient gels (Life Technologies) and transferred to polyvinylidene difluoride membrane (Immobilon-P, Merck Millipore) at 150 V in 1× MOPS transfer buffer (Life Technologies). Blots were blocked in Tris-buffered saline (TBS) containing 5% skim milk powder, probed with primary antibody [mouse α Myc (9B11; 1/5,000, Cell Signalling Technology), rabbit α HA (1/500, Cell Signalling Technology), mouse α β-actin (AC-15; 1/20,000, Cell Signalling Technology), rabbit α EGFR (1/2,000, Cell Signalling Technology), mouse α Na^+^/K^+^ ATPase (alpha 1; clone C464.6; 1/4,000, Merck Millipore), rabbit α SK2 (c-39; 1/2,000, gift of J. Adelman)] followed by incubation with horseradish peroxidase-conjugated goat anti-rabbit or mouse IgG (1/20,000, Biorad) and detection by SuperSignal West Pico or FEMTO chemiluminescent substrate (Pierce). Blots were scanned using the Odyssey Infrared Imaging System (Li-cor) and densitometry analysis was carried out using Image Studio Lite software.

All data are expressed as mean ± standard error of the mean (SEM). Statistical analysis was performed using GraphPad Prism (GraphPad Software). All the data were tested for normal distribution using a normality test. If data were normally distributed, a Student’s unpaired *t*-test was used. If the data did not pass the normality test, the Mann-Whitney test was used. Significance was determined at *p* < 0.05.

### Co-immunoprecipitation

Supernatants were precleared for 1 h at 4°C with 75 μl of 50% Sepharose bead slurry (Amersham) and 1.5 μg of species-specific IgG (Sigma, 1 mg/ml). Protein G sepharose bead slurry and mouse IgG were used for the samples immunoprecipitated with mouse α Myc (9B11; Cell Signalling Technology); protein A sepharose bead slurry and rabbit IgG for the samples immunoprecipitated with the rabbit α HA (Cell Signalling Technology) antibody. Samples were then spun at 13,000 rpm for 5 min and the resulting supernatants were incubated overnight at 4°C respectively with mouse α Myc antibody (2 μl/1.5 mg proteins) or rabbit α HA (1:50) antibodies. The following day, the α Myc samples were incubated with 75 μl of 50% protein G sepharose beads and the α HA samples with 75 μl of 50% protein A sepharose beads for a further 3 h at 4°C. Samples were centrifuged at 3,000 rpm for 5 min at 4°C and the recovered pellets were washed three times in RIPA buffer [150 mM NaCl, 1% Triton X-100, 0.5% sodium deoxycholate, 0.1% SDS, 50 mM Tris pH = 8.0 and protease inhibitors (Roche)]. Samples were eluted with sample buffer containing DTT and denatured by boiling for 5 min. Co-immunoprecipitation samples (IP) were run together with the input (IN) and the supernatant (S) resulting from the immunoprecipitation, transferred as described above and blotted with mouse α Myc and rabbit α HA as described above.

### Lentiviral Constructs

**Figure 1:**
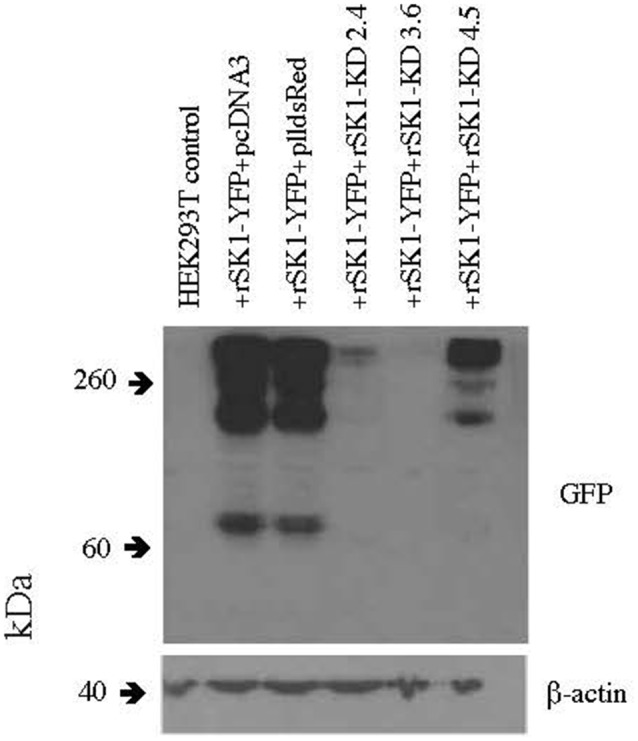
rSK1-KD 3.6 reduces rSK1-YFP expression in HEK293T cells. Western blot of total cell lysates of HEK293T cells transfected with rSK1-YFP and co-transfected with pcDNA3 or plldsRed or three different knock-down hairpin constructs: rSK1-KD 2.4 or rSK1-KD 3.6 or rSK1-KD 4.5. rSK1-KD 3.6 reduced the expression of rSK1-YFP by over 90% and was used in all further experiments. β-actin was used as a loading control.

Several knock-down hairpin constructs were produced using annealed phosphorylated oligonucleotides, which were ligated into the pll3.7 dsRed vector digested with *HpaI* and *XhoI*. The pll3.7 dsRed vector has the EGFP of pll3.7 vector replaced with dsRed. HEK293T cells were plated at a density of 5 × 10^5^ cells per 35 mm well and were transfected 2 h later using Lipofectamine 2,000 (Life Technologies) as per the manufacturer’s instructions with constructs expressing rat SK1-YFP (gift of G. Moss) and three different plasmids carrying SK1 knock down hairpins (rSK1-KD 2.4, 3.6 and 4.5) at a ratio of 1:1. Forty-eight hours later, the cells were checked for YFP and dsRed expression, washed with PBS and lysed in 600 μl 1× sample buffer. Thirty microliters of this was run on an SDS-PAGE gel, transferred and western blots carried out using rabbit α GFP (1/500, Merck Millipore) primary antibody as per methods above except that x-ray film was used for detection. Mouse β-actin (AC-15; 1/20,000, Cell Signalling Technology) was used as a loading control. Out of the three plasmids, rSK1-KD 3.6 was the most successful at reducing SK1-YFP expression (Figure 1). The primers used to produce this knockdown construct are as follows–

- pLLrSK1_3S: tgtctcatagcccaagccatatttcaagagaatatggcttgggctatgagacttttttc
- pLLrSK1_3AS: tcgagaaaaaagtctcatagcccaagccatattctcttgaaatatggcttgggctatgagaca

To enable us to determine the virally infected neurons, we replaced the CMV promoter (expressing the dsRed) in pll3.7 dsRed with the hSyn promoter using the Not and Nhe restriction sites and the following primers–

- Synapsin400-Not-F: aaagcggccgcgtgtctagactgcagagggccct
- Synapsin400-Nhe-R: aaagctagcttctcgactgcgctctcagg

A scrambled (SCRAM) control plasmid was also produced using a random combination of the hairpin sequence of the successful knockdown construct. The primers used to produce the SCRAM construct are as follows–

- SCRAM pLLrSK1_3S: tggcactttaacgccaatcactttcaagagaagtgattggcgttaaagtgccttttttc
- SCRAM pLLrSK1_3AS: tcgagaaaaaaggcactttaacgccaatcacttctcttgaaagtgattggcgttaaagtgcca

The rat SK2 overexpression plasmid pJPA5.rSK2.Myc3 (gift of J. Adelman) has a triple Myc tag located extracellularly between the S3 and S4 domains. The Myc-tagged SK2 was further cloned into the lentiviral FUGW plasmid (gift of P. Osten) using the AgeI and EcoRI restriction sites and the following primers–

- F-rSK2: gggaccggtgaaatagccatgagcagctgcaggtacaacggg
- R-rSK2: ggggaattcgctactctcagatgaagttgg

The rat SK1 overexpression plasmid pcDNA3.1-rSK1_HA (gift of G. Moss) has a HA tag located intracellularly between the S2 and S3 domains. The HA-tagged SK1 was further cloned into the lentiviral FUGW plasmid (gift of P. Osten).

For lentivirus production, all plasmids (pMDG, pMDL g/p RRE, pRSV Rev and the pll3.7 transfer vector) were prepared using the Qiagen Endofree maxiprep kit and lentivirus was prepared via calcium phosphate transfection of 80% confluent triple T175 flasks of HEK293T cells. Cells were grown in DMEM (Life Technologies) plus 10% FBS and 1% penicillin/streptomycin (Life Technologies). Forty microgram of transfer vector together with 20 μg of the other three plasmids were transfected per triple T175 flask. Plasmids were diluted to 0.5 μg/ml in TE buffer and made up to a volume of 3 ml with sterile distilled water. Three-hundred microliter of 2.5 M CaCl_2_ was added together with 3 ml of 2× BBS (50 mM BES, 280 mM NaCl, 1.5 mM Na_2_HPO_4_, pH = 6.95). This was incubated for 20 min at room temperature before adding the mix dropwise to the cells. The cells were incubated for 3–4 h at 37°C plus 5% CO_2_ before the removal of the calcium phosphate mix and addition of new media. Viral supernatants were removed 48 h post-transfection, filtered using a 0.45 μm filter unit and then centrifuged for 4 h at 20,000 *g* over a 20% sucrose cushion. The resulting pellet was resuspended overnight in PBS containing 1% BSA and then ultracentrifuged at 44,000 *g* for 90 min to concentrate the virus. The virus pellet was resuspended in PBS containing 1% BSA.

Neuronal cultures were infected at 4–5 DIV and the virus was left on for 4–12 h. The virus was then removed and conditioned media added to the cultures. Biotinylation experiments and quantitative PCR (qPCR) were undertaken 4–5 days post infection. Knockdown and overexpression lentiviral constructs were also tested for specificity and expression using qPCR. Briefly, RNAs were purified using a RNeasy kit (Qiagen), DNase 1 (Qiagen) treated and reverse transcribed to produce complementary DNA using random hexamers and the SuperScript III First Strand Synthesis System (Life Technologies), as per the manufacturer’s instructions. Standard qPCR was carried out on a Rotorgene RG-3000 Thermocycler using the Platinum SYBR Green qPCR UDG SuperMix (Life Technologies) and 0.2 μM of the following primers:

- rSK1 Fwd—tca tct cca tta cct tcc tg
- rSK1 Rev—agc ctg gtg tgt ttg tag at
- rSK2 Fwd—gtc gct gta ttc ttt agc tct g
- rSK2 Rev—acg ctc ata agt cat ggc
- rGAPDH Fwd—gag tct act ggc gtc ttc ac
- rGAPDH Rev—cca tcc aca gtc ttc tga gt

Reactions were performed in either duplicate or triplicate. The PCR program was as follows: 50°C 2 min, 95°C 10 min, 35 cycles −95°C 10 s, 54°C 15 s, 72°C 20 s. Relative expression of rSK1 and rSK2 mRNA was obtained using the comparative Ct method.

### Cosm6 Transfections

For the Cosm6 transfections- pJPA5.rSK2.Myc3 and pcDNA3.1-rSK1_HA along with pcDNA3 for the single construct transfections were transfected using the same protocol as described above but using a ratio of 1:2:1.

### Immunohistochemistry

Hippocampal cultures were washed in PBS and fixed in 4% PFA containing 4% sucrose. After three washes in PBS, the cells were first blocked in a 0.3% BSA/0.1% Triton X-100 solution, then incubated in primary antibody overnight at 4°C, washed in PBS and left in secondary antibody (Life Technologies) for a further 1 h at room temperature. Stained cultures were analyzed on a confocal fluorescence microscope (Zeiss LSM510).

### Electrophysiology

Rat hippocampal neuronal cultures were recorded at least 2 weeks after viral infection (DIV 15–17). Coverslips were transferred from the incubator to the recording chamber, located on a stage of a fluorescence microscope (Zeiss 710). Ringer solution (140 mM NaCl, 5 mM KCl, 2 mM CaCl_2_, 1 mM MgCl_2_ 10 mM glucose and 10 mM HEPES) was constantly perfused at 1–1.5 ml/min at room temperature. Whole-cell recordings were obtained from dsRed-positive neurons (SCRAM or SK1 KD) or neurons (rSK1-HA and rSK2-myc) using a K-methyl-sulfate based internal solution containing (mM): 135 KMeSO_4_, 5 NaCl, 10 Hepes, 2 Mg_2_-ATP, 0.3 Na_3_-GTP, 0.1 spermine and 7 phosphocreatine (pH = 7.3, ~290 mOsmol).

Tetrodotoxin (TTX, 1 μM, Sigma), D-(−)-2-Amino-5-phosphonopentanoic acid (D-APV, 20 μM, Tocris) and 6-cyano-7-nitroquinoxaline-2,3-dione disodium salt (CNQX, 20 μM, Tocris) were added to the ringer solution to reduce spontaneous activity and isolate mAHP and I_AHP_. For a recording of mAHP, cells were injected with a 400 pA/100 ms step either at resting membrane potential or by clamping the cells at −50 mV. For recordings of I_AHP_, cells were held at −50 mV and a depolarizing step (+60 mV) to +10 mV for 100 ms was used to increase Ca^2+^ influx through voltage-gated Ca^2+^channels, which are necessary to activate I_AHP_ (Stocker et al., 2004).

Data were collected using Axograph X software and a Multiclamp 700B amplifier (Molecular Devices). Signals were filtered at 10 kHz and digitized at 50 kHz using an ITC-16 A/D converter (InstruTech).

## Results

### rSK1 and rSK2 Co-assemble in Cosm6 Cells

**Figure 2:**
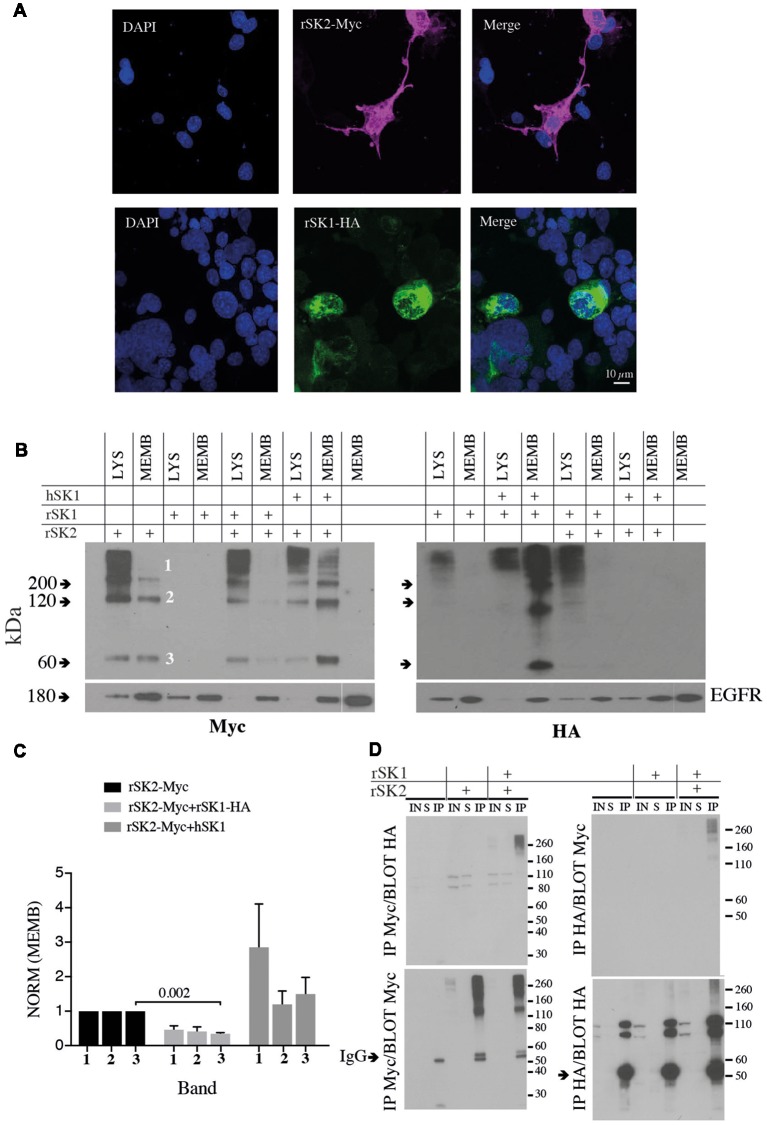
rSK1 regulates the membrane expression of rSK2 channels. **(A)** Immunocytochemistry of Cosm6 cells transfected with rSK2-Myc or rSK1-HA. rSK2-Myc (pink), rSK1-HA (green), DAPI (blue). **(B)** Immunoblots from Cosm6 cells transfected with rSK1-HA, rSK2-Myc and hSK1 in different combinations. Shown are the total lysate (LYS) and membrane (MEMB) fraction isolated using surface biotinylation probed with either Myc (rSK2; left) or HA (rSK1; right) antibody. EGFR was used as the loading control. rSK2 transfection results in three size bands that represent monomeric protein (band 3, ~60 kDa), dimers (band 2, ~120 kDa) and tetramers (band 1, >200 kDa). Co-transfection of rSK2 and rSK1 reduced the amount of rSK2 expressed in the cell membrane. **(C)** Bar graphs show normalized relative density of SK2, as described in the “Materials and Methods” section, in the membrane fraction from Cosm6 cells transfected with rSK2 alone or co-transfected with rSK1 or hSK1. Co-transfection with rSK1-HA significantly reduced the 60 kDa band (*p* = 0.002). Co-transfection with hSK1 increased expression of rSK2. **(D)** Co-immunoprecipitation (co-IP) assay of transfected Cosm6 cells shows co-assembly of rSK1-rSK2. Solubilized protein from Cosm6 cells transfected with rSK1 and/or rSK2 was immunoprecipitated with Myc (left) or HA (right) and immunoblotted with either Myc or HA as indicated.

To test expression of SK channels, rSK1 and rSK2 were epitope-tagged with HA and Myc, respectively (see “Materials and Methods” section). As shown previously (D’Hoedt et al., 2004; Church et al., 2015), transfection in Cosm6 cells shows that rSK2 was expressed throughout the cell, while rSK1-HA was restricted to the cytosolic somatic compartment (Figure 2), likely the endoplasmic reticulum and Golgi (Church et al., 2015). As shown by others (Strassmaier et al., 2005), western blot analysis using Myc and HA antibodies shows bands of several sizes that correspond to monomeric protein (~60 kDa, band 3), dimers (~120 kDa, band 2) and a large molecular band (~200 kDa, band 1), which is likely to be a tetramer (Figure 2B). Separation of the membrane fraction (MEMB on blots) using surface biotinylation (see “Materials and Methods” section) shows that unlike rSK2, rSK1 channels are not detectable on the plasma membrane. When rSK1 and rSK2 were co-expressed, again rSK2 was detectable in the membrane fraction but rSK1 was not (Figure 2B), but the level of rSK2 protein in the membrane fraction was reduced (Figures 2B,C). There was a clear reduction in monomeric rSK2 (*n* = 3, *p* = 0.002), but other higher molecular weight species were also greatly reduced (Figure 2C). In contrast to rSK1, hSK1 is known to express as functional channels (D’Hoedt et al., 2004), and forms heteromultimeric channels (Ishii et al., 1997; Benton et al., 2003; Church et al., 2015). In agreement, co-expression of hSK1 with the rSK2 increased the amount of rSK2-Myc on the cell membranes (Figures 2B,C).

What explains the reduction in membrane rSK2 when it is co-expressed with rSK1? As rSK1 is retained in cytosolic compartments and not trafficked to the membrane (Benton et al., 2003; Church et al., 2015), it is possible that rSK2 co-assembles with rSK1 with the resulting heteromultimers being retained, leading to a reduction of membrane rSK2. We, therefore, tested for interactions between these two channels using co-immunoprecipitation (co-IP). rSK1-HA and rSK2-Myc were expressed in Cosm6 cells, and Myc or HA antibodies used to isolate rSK2 or rSK1 using sepharose A/G beads followed by immunoblot analysis of SK protein. IP of Cosm6 cell homogenates using anti-Myc antibody revealed the presence of rSK2 in both supernatant, input and co-immunoprecipitated samples, from single transfected (rSK2-Myc) and co-transfected cells (rSK2-Myc+rSK1-HA; Figure 2D). Probing with HA antibody revealed the presence of rSK1-HA in the multimeric complex (Figure 2D, left). Complimentary experiments using the anti-HA antibody to precipitate rSK1 protein lysates and probing with anti-Myc also revealed the presence of rSK2 in the multimeric high molecular weight band (Figure 2D, right). It was notable that following IP with myc and probe with HA or the reverse, while very high molecular weight (>200 kD) complexes are present, there is a dearth of complexes at ~110 kD (Figure 2D, upper panels). We interpret this to show the presence of tetrameric heteromeric protein.

### Overexpression of rSK1 in Neurons Decreases Plasma Membrane rSK2

These results show that when expressed in Cosm6 cells rSK1 and rSK2 can co-assemble. Moreover, the interaction between the two channels affects the total rSK2 present in the cell membrane. Thus, rSK1 channels appear to “trap” rSK2 in the cytoplasmic compartment, therefore reducing the total amount of rSK2 trafficked to the cell membrane.

**Figure 3:**
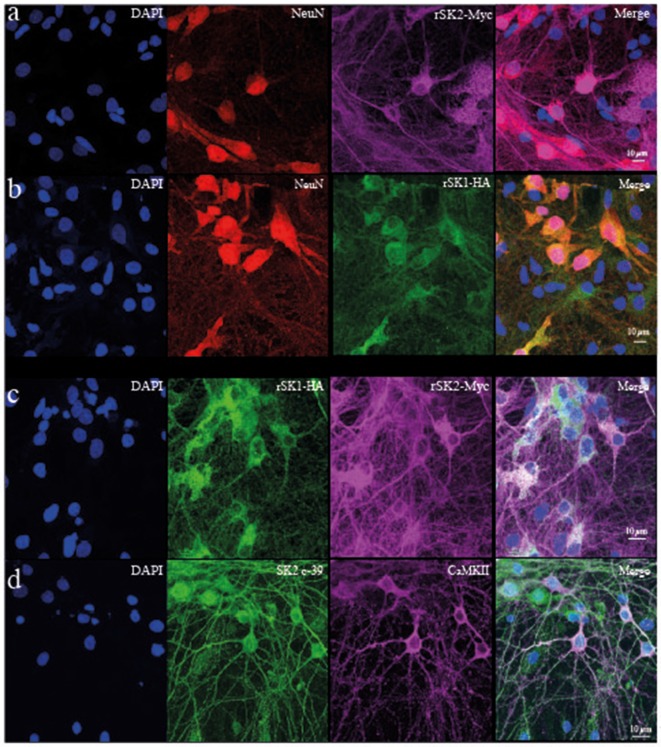
rSK1 and rSK2 expressed in rat hippocampal neuronal cultures. Cultured hippocampal neurons were transduced with rSK2-Myc **(A)** or rSK1-HA **(B)** or both **(C)** using lentivirus and immunostained for DAPI (blue), the neuronal marker NeuN (red), Myc (purple) or HA (green). **(A,B)** SK2 (Myc) and SK1 (HA) are expressed throughout the soma and processes of transduced neurons. DAPI **(C)** co-expression of rSK2 and rSK1 shows that both proteins are present in the same compartments. **(D)** Immunocytochemistry using a specific SK2 antibody (c-39; green) shows that endogenous rSK2 is present in the soma and throughout the processes of cultured neurons. Hippocampal neurons are marked using CaMKII (purple).

To test if rSK1 acts similarly in neurons, we turned to cultured rat hippocampal neurons. rSK1-HA and rSK2-Myc were delivered using lentivirus (see “Materials and Methods” section). We first tested the effect of overexpression of rSK1 or rSK2 on mRNA levels, using qPCR. Increasing the volume of rSK1 or rSK2 expressing virus added to neuronal cultures increased the relative expression of mRNA of the specific gene delivered without affecting the other: infection of 1 × 10^5^ rat hippocampal cultures with 0.2 μl and 0.5 μl rSK1-HA lentivirus increased rSK1 relative expression by 60 and 200-fold, respectively, while infection with 0.2 μl and 0.5 μl of rSK2-Myc lentivirus increased rSK2 relative expression by 10 and 40-fold, respectively (data not shown). Next, expression of SK2 protein was tested using immunocytochemistry (Figure 3). Transduction of rSK2-Myc (Figure 3A) or rSK1-HA (Figure 3B) showed clear expression of Myc and HA in cells positive for the neuronal marker NeuN. Co-expression of rSK1 and rSK2 show that the two are at least partially co-localized in neurons (Figure 3C), consistent with heterodimer formation of rSK1 and rSK2. It can also be seen that in neurons when rSK1-HA is expressed alone (Figure 3B), its distribution is not confined to the soma, but spreads throughout the cell, consistent with the expression of endogenous SK2 in these neurons (Figure 3D).

**Figure 4:**
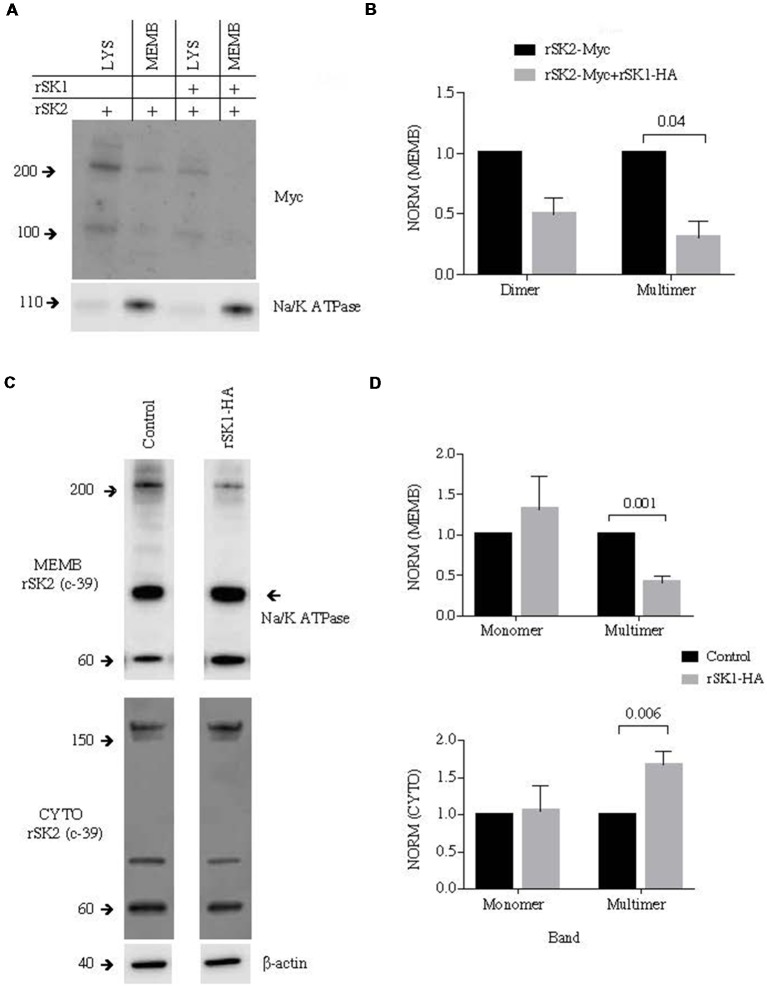
rSK1 regulates membrane expression of rSK2 channels in cultured neurons. Cultured hippocampal neurons were transduced to express rSK1-HA or rSK2-Myc or both. Surface protein was biotinylated, and solubilized protein from neurons separated into total lysate (LYS) and membrane fraction (MEMB) as detailed in the “Materials and Methods” section. Immunoblots were probed using the Myc antibody to test for rSK2 expression. Na/K ATPase or β-actin was used as loading controls. **(A)** Co-expression of rSK1 and rSK2 reduces membrane expression of rSK2. **(B)** Graph shows normalized relative density of SK2, in the membrane fraction from neurons transduced with rSK2 alone or rSK2 and rSK1. Co-infection significantly reduced the band at 200 kDa (*p* = 0.04). **(C)** Expression of rSK1 in neurons reduced surface expression of endogenous rSK2. Cultured neurons were transduced to express rSK1 using lentivirus. Blots show solubilized protein from the membrane fraction (upper blots) and cytosolic fraction (lower blots) from control neurons (left) and neurons transduced with rSK1(right). Blots were probed with anti SK2 antibody (c-39). **(D)** Quantification of SK2 protein expression in the membrane (upper graph) and cytosolic (lower graph) fraction from control neurons and neurons transduced with rSK1. Neurons infected with rSK1 had a significantly lower amount of endogenous high molecular weight rSK2 in the membrane fraction and this was balanced by an increase in the total cytoplasmic fraction.

To test for the presence of rSK2 in the plasma membrane we again turned to the surface biotinylation assay to separate the membrane compartment. rSK1-HA and rSK2-Myc were transduced using lentivirus. As in Cosm6 cells (Figure 2), we found that co-expression of rSK2 with rSK1 decreased the amount of rSK2 detected in the membrane fraction of infected neurons (Figures 4A,B). This reduction was particularly significant in the high molecular weight band, again suggesting delivery of a heteromultimeric protein (*n* = 3, *p* = 0.04; Figure 4B). To account for possible effects of overexpressing rSK2, we tested the impact of rSK1 expression on endogenous rSK2 using the specific SK2 antibody (c-39). When rSK1-HA was transduced in hippocampal neurons, the endogenous levels of rSK2 protein detected in the cell membrane was reduced (Figure 4C, upper panel), while the cytoplasmic fraction increased (Figure 4C, bottom panel). Quantification of the relative density of endogenous rSK2 (c-39) normalized to β-actin shows that the density of the high molecular band was significantly reduced in the membrane fraction in comparison to uninfected neurons (*n* = 5, *p* = 0.001; Figure 4D), but was increased in the cytosolic fraction (*n* = 4, *p* = 0.006; Figure 4D, bottom panel). This increase in SK2 in the “cytoplasmic” fraction is likely due to the fact that rSK1-rSK2 heteromultimers are not trafficked to the plasma membrane, but remain trapped in the cytoplasmic compartment, possibly the endoplasmic reticulum.

### rSK1 Modulates Functional SK Channels

**Figure 5:**
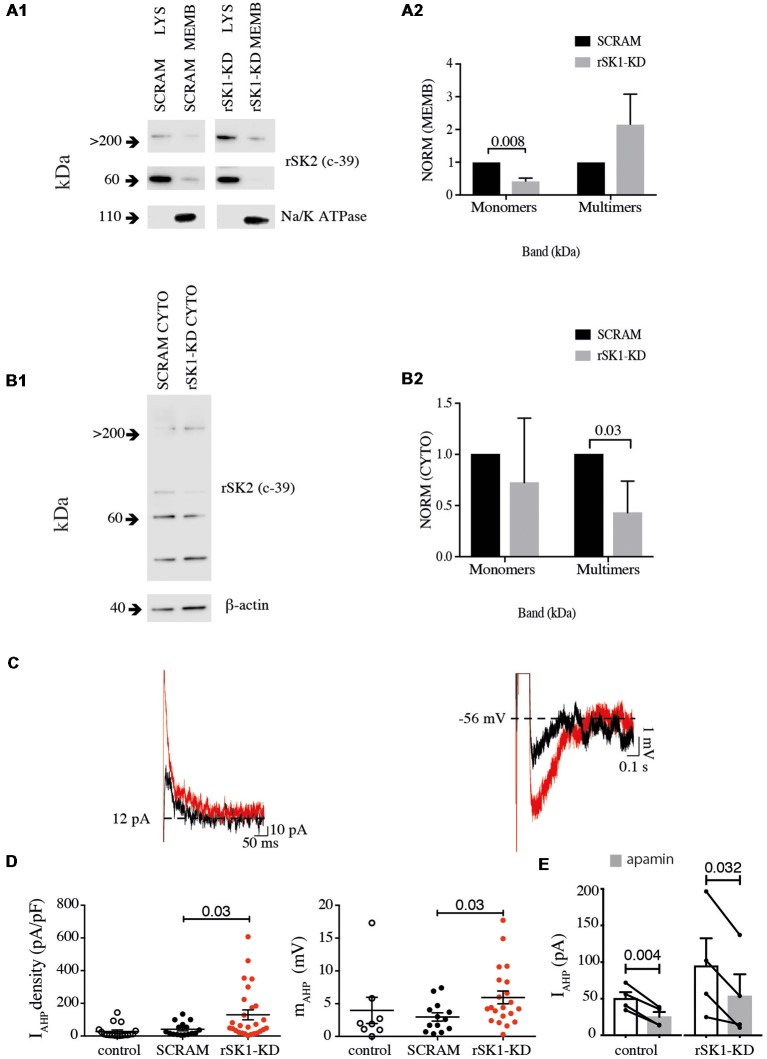
Knock down of rSK1 enhances I_AHP_ in hippocampal neurons. Cultured hippocampal neurons were transduced to express a hairpin knockdown vector for SK1 (rSK1-KD) or a scrambled control vector (SCRAM). Surface protein was biotinylated, and solubilized protein from neurons separated into total lysate (LYS) and membrane fraction (MEMB) as detailed in the “Materials and Methods” section. Blots were immunoprobed using the rSK2 antibody (c-39). **(A1)** Knockdown of SK1 protein results in an overall reduction of total endogenous monomeric SK2 protein, but enhancement of high molecular weight SK channels in the plasma membrane. Na^+^/K^+^ ATPase was sued as the loading control. **(A2)** Normalized relative density of endogenous rSK2 in the membrane fraction [NORM (MEMB)] from neurons infected with rSK1-KD. rSK1-KD significantly decreased the amount of endogenous monomeric rSK2, but increased high molecular weight rSK2. Na^+^/K^+^ ATPase (~110 kDa) was used to normalize all the loaded samples for the MEMB fraction. **(B1)** Knockdown of rSK1 results in a reduction in high molecular weight SK2 in the cytoplasmic fraction. **(B2)** Quantification of blot shown in B1. β-actin (~40 kDa) was used to normalize all the loaded samples for the CYTO fraction. **(C)** Knockdown of rSK1 in hippocampal neurons (red traces) increased the amplitude of the I_AHP_ in voltage clamp (left) and the medium AHP in current clamp (right) in comparison to SCRAM infected neurons (traces in gray). **(D)** Quantification of the impact of the increase in the I_AHP_ current shown as pA/pF and the medium AHP following knockdown of rSK1. **(E)** The enhanced I_AHP_ current following knockdown of rSK1 is apamin (100 nM) sensitive consistent with SK2 channels.

In mammalian neurons, SK channels contribute to the medium duration AHP that follows action potentials (I_AHP_), and in some types of neurons they are also present at excitatory synapses where they modulate synaptic strength (Faber et al., 2005; Ngo-Anh et al., 2005; Adelman et al., 2012). We have shown that overexpression of rSK1 seems to reduce the amount of membrane rSK2 protein. We next tested if altering endogenous rSK1 levels would have an impact on functional membrane rSK channels in neurons. To disrupt endogenous rSK1, we used short hairpin RNA interference (RNAi) constructs to reduce rSK1 expression in hippocampal neurons using lentivirus (see “Materials and Methods” section). rSK2 protein levels in the membrane were assessed using the biotinylation assay and western blot analysis (Figures 5A,B). Knockdown of rSK1 (rSK1-KD) significantly reduced the amount of rSK2 monomers (60 kDa) expressed in the cell membrane in comparison to neurons infected with scrambled virus (*n* = 4, *p* = 0.008), but the total membrane rSK2 tetramers (>200 kDa) appeared to be higher than the scrambled control (Figure 5A2). In the cytosolic compartment, high molecular weight (>200 kDa) rSK2 protein was significantly reduced as compared to scrambled control cells (*n* = 4, *p* = 0.03; Figure 5B2).

To test for functional expression of rSK channels, whole-cell recordings were obtained from infected hippocampal neurons in culture (Figures 5C,D). The passive membrane properties of neurons in infected with SK2 RNAi hairpins and scrambled controls were not different, and are given in Supplementary Table S1. Reducing rSK1 with RNAi increased the amplitude of I_AHP_ in comparison to that in scrambled infected neurons (*p* = 0.03; Figure 5C). As expected, switching to current clamp also revealed an increase in the amplitude of the medium AHP (*p* = 0.03; Figure 5D), and this enhanced I_AHP_ current was blocked by apamin (Figure 5E). Together, these results support a role for rSK1 channels in regulating the expression of rSK2 on the cell membrane.

## Discussion

SK1, SK2 and SK3 encode calcium-activated potassium channels that are widely expressed in the mammalian brain. In rodents, rSK1 and rSK2 are expressed in strongly overlapping distributions. However, while expression of rSK2 channels produces functional calcium-activated potassium channels, rSK1 channels are made but not trafficked to the plasma membrane (D’Hoedt et al., 2004; Church et al., 2015), and the functional role of these channels is not clear. In this study, we show that rSK1 channels co-assemble with rSK2, and regulate the plasma membrane levels of rSK2. Thus, expression of rSK1 in neurons leads to the formation of heteromultimers containing rSK2 that are trapped in the cytoplasmic compartment and a reduction in the total rSK2 in the plasma membrane. In support of this, knockdown of endogenous rSK1 in cultured neurons results in an increase in membrane rSK2. These results suggest that rSK1 channels, rather than functioning as ion channels, are involved in regulating the expression of rSK2 channels in the plasma membrane.

While SK channels were cloned more than 20 years ago, antibodies for immunoprecipitation and immunohistochemistry for all three members are not easily available, thus we have used epitope-tagged channels to study their expression and trafficking. Here using rSK1 tagged with HA (rSK1-HA) and rSK2 tagged with Myc (rSK2-Myc), in agreement with previous studies (D’Hoedt et al., 2004; Church et al., 2015), we show that when expressed in Cosm6 cells, rSK2 channels are present in the plasma membrane, rSK1 channels are made but not trafficked from the cytoplasmic compartment, with little or no protein detectable in the membrane compartment. When rSK1 and rSK2 are co-expressed in Cosm6 cells, we show using co-immunoprecipitation that these channels co-assemble and there is a significant reduction in the amount of rSK2 in the plasma membrane. It is well known that hSK1 channels can form functional channels that are expressed on the cell surface (D’Hoedt et al., 2004; Church et al., 2015). In agreement with this, co-expression of rSK2 with hSK1 had an opposite effect to that of rSK1 with an increase in membrane rSK2.

These results in Cosm6 cells were confirmed in rat hippocampal neurons. Co-expression of rSK1-HA and rSK2-Myc resulted in an overall reduction in the amount of rSK2-Myc trafficked to the membrane. Importantly, expression of exogenous rSK1-HA in cultured neurons also resulted in a reduction in the total endogenous rSK2, showing that expression of rSK1 can modify the levels of membrane rSK2 channel. Finally, we show that by reducing the amount of rSK1 channels using RNAi, increases the amount of endogenous rSK2 channels expressed in the cell membrane, and is consistent with the finding that transgenic mice lacking SK1 show no overall effects on hippocampal neuronal electrophysiology (Bond et al., 2004). We show that reducing the rSK1 content of hippocampal neurons reduces the overall amount of rSK2 monomeric protein in the membrane but increases the total large molecular weight fraction (Figure 5). This is perhaps expected as we have not modified the expression level of rSK2 protein, and as rSK1 levels are lower, more channels are trafficked to the membrane where they co-assemble as high molecular weight homomeric multimers. As a result, total monomeric protein levels are lower. This increase in membrane rSK2 levels has a functional impact as it also increases the amplitude of the SK-mediated I_AHP_ in infected rat neurons. Thus, by increasing or reducing the amount of rSK1 channels expressed in a heterologous system or rat neurons, we can decrease or increase rSK2 channels expressed on the cell membrane.

Previous studies have shown that when expressed in HEK293 cells, the interaction of rSK1 and rSK2 channels resulted in an overall larger SK-channels mediated current and a change in their pharmacology (Benton et al., 2003; Church et al., 2015). This appears to be in contrast with our results in both Cosm6 cells and rat neurons where overexpression of rSK1-HA reduced the amount of rSK2 channels expressed in the cell membrane. However, it remains possible that these channels assemble as heteromultimers with the total fraction of SK1-SK2 heteromeric protein in the membrane being very low.

Our results suggest that in the rodent brain rSK1 channels, rather than acting as independent membrane ion channels, are involved in trafficking of rSK2 channels to the plasma membrane. hSK1, which is ~84% homologous to the rodent channels (D’Hoedt et al., 2004), behaves entirely differently, being translated as a functional ion channel (Church et al., 2015). The reason for this difference is not clear. It is possible, that the rSK1-rSK2 interaction and the regulation of rSK2 channels expressed on the cell membrane is important during development. rSK1 and rSK2 channel transcripts within the hippocampus (CA1, CA3 and dentate gyrus) start being expressed at embryonic day 19 (E19) in rodents (Gymnopoulos et al., 2014), with their expression patterns being very similar, showing colocalization (Stocker and Pedarzani, 2000). These similar expression patterns could indicate a developmental change within the expression of rSK2 channels on the cell membrane that could affect neuronal physiology. Interestingly, rSK2 was found to be present mainly in the ER of CA1 pyramidal neurons at P5. By P30, however, most rSK2 was present at spines and dendrites changing the cellular physiology (Ballesteros-Merino et al., 2012). It is, therefore, possible that rSK1 channels modulate the amount of rSK2 channels expressed on the cell membrane. While rSK2 channels are trapped in the ER by rSK1 channels, neurons can receive more inputs and increased the excitation in the early developmental stages, which will increase the memory acquisition. Once later developmental stages are reached, however, rSK1 channels may have a different role in regulating the expression of rSK2 channels and the neuronal physiology.

## Ethics Statement

This was approved by the University of QLD Ethics Committee.

## Author Contributions

EA discussed experiments, did experiments, and wrote the manuscript. PSe did experiments and wrote the manuscript. LX made virus. MR did experiments and made figures. AT did experiments, made figures and wrote the manuscript. PSa designed experiments, made figures and wrote the manuscript.

## Conflict of Interest Statement

The authors declare that the research was conducted in the absence of any commercial or financial relationships that could be construed as a potential conflict of interest.

## References

[B1] Adelman J. P. Maylie J. Sah P. (2012). Small-conductance Ca^2+^-activated K^+^ channels: form and function. Ann. Rev. Physiol. 74, 245–269. 10.1146/annurev-physiol-020911-153336 21942705

[B2] Ballesteros-Merino C. Lin M. Wu W. W. Ferrandiz-Huertas C. Cabanero M. J. Watanabe M.. (2012). Developmental profile of SK2 channel expression and function in CA1 neurons. Hippocampus 22, 1467–1480. 10.1002/hipo.20986 22072564 PMC3359419

[B3] Benton D. C. Monaghan A. S. Hosseini R. Bahia P. K. Haylett D. G. Moss G. W. (2003). Small conductance Ca^2+^-activated K^+^ channels formed by the expression of rat SK1 and SK2 genes in HEK 293 cells. J. Physiol. 553, 13–19. 10.1113/jphysiol.2003.054551 14555714 PMC2343499

[B4] Bond C. T. Herson P. S. Strassmaier T. Hammond R. Stackman R. Maylie J.. (2004). Small conductance Ca^2+^-activated K^+^ channel knock-out mice reveal the identity of calcium-dependent afterhyperpolarization currents. J. Neurosci. 24, 5301–5306. 10.1523/jneurosci.0182-04.2004 15190101 PMC2831645

[B5] Church T. W. Weatherall K. L. Correa S. A. Prole D. L. Brown J. T. Marrion N. V. (2015). Preferential assembly of heteromeric small conductance calcium-activated potassium channels. Eur. J. Neurosci. 41, 305–315. 10.1111/ejn.12789 25421315

[B6] Coetzee W. A. Amarillo Y. Chiu J. Chow A. Lau D. McCormack T.. (1999). Molecular diversity of K^+^ channels. Ann. N Y Acad. Sci. 868, 233–285. 10.1111/j.1749-6632.1999.tb11293.x 10414301

[B7] Delaney A. J. Sedlak P. L. Autuori E. Power J. M. Sah P. (2013). Synaptic NMDA receptors in basolateral amygdala principal neurons are triheteromeric proteins: physiological role of GluN2B subunits. J. Neurophysiol. 109, 1391–1402. 10.1152/jn.00176.2012 23221411

[B8] Desai R. Peretz A. Idelson H. Lazarovici P. Attali B. (2000). Ca^2+^-activated K^+^ channels in human leukemic Jurkat T cells. Molecular cloning, biochemical and functional characterization. J. Biol. Chem. 275, 39954–39963. 10.1074/jbc.m001562200 10991935

[B9] D’Hoedt D. Hirzel K. Pedarzani P. Stocker M. (2004). Domain analysis of the calcium-activated potassium channel SK1 from rat brain. Functional expression and toxin sensitivity. J. Biol. Chem. 279, 12088–12092. 10.1074/jbc.c300382200 14761961

[B10] Faber E. S. Delaney A. J. Sah P. (2005). SK channels regulate excitatory synaptic transmission and plasticity in the lateral amygdala. Nat. Neurosci. 8, 635–641. 10.1038/nn1450 15852010

[B11] Fiorillo C. D. Williams J. T. (1998). Glutamate mediates an inhibitory postsynaptic potential in dopamine neurons. Nature 394, 78–82. 10.1038/27919 9665131

[B12] Gymnopoulos M. Cingolani L. A. Pedarzani P. Stocker M. (2014). Developmental mapping of small-conductance calcium-activated potassium channel expression in the rat nervous system. J. Comp. Neurol. 522, 1072–1101. 10.1002/cne.23466 24096910 PMC4016743

[B13] Ishii T. M. Maylie J. Adelman J. P. (1997). Determinants of apamin and d-tubocurarine block in SK potassium channels. J. Biol. Chem. 272, 23195–23200. 10.1074/jbc.272.37.23195 9287325

[B14] Joiner W. J. Wang L.-Y. Tang M. D. Kaczmarek L. K. (1997). hSK4, a member of a novel subfamily of calcium-activated potassium channels. Proc. Natl. Acad. Sci. U S A 94, 11013–11018. 10.1073/pnas.94.20.11013 9380751 PMC23566

[B15] Köhler M. Hischberg B. Bond C. T. Kinzie J. M. Marrion N. V. Maylie J.. (1996). Small-conductance, calcium-activated potassium channels from mammalian brain. Science 273, 1709–1714. 10.1126/science.273.5282.1709 8781233

[B16] Lee C. H. MacKinnon R. (2018). Activation mechanism of a human SK-calmodulin channel complex elucidated by cryo-EM structures. Science 360, 508–513. 10.1126/science.aas9466 29724949 PMC6241251

[B17] Lin M. T. Luján R. Watanabe M. Adelman J. P. Maylie J. (2008). SK2 channel plasticity contributes to LTP at Schaffer collateral-CA1 synapses. Nat. Neurosci. 11, 170–177. 10.1038/nn2041 18204442 PMC2613806

[B18] Ngo-Anh T. J. Bloodgood B. L. Lin M. Sabatini B. L. Maylie J. Adelman J. P. (2005). SK channels and NMDA receptors form a Ca^2+^-mediated feedback loop in dendritic spines. Nat. Neurosci. 8, 642–649. 10.1038/nn1449 15852011

[B19] Sah P. (1996). Ca^2+^-activated K^+^ currents in neurones: types, physiological roles and modulation. Trends Neurosci. 19, 150–154. 10.1016/s0166-2236(96)80026-9 8658599

[B20] Sailer C. A. Hu H. Kaufmann W. A. Trieb M. Schwarzer C. Storm J. F.. (2002). Regional differences in distribution and functional expression of small-conductance Ca^2+^-activated K^+^ channels in rat brain. J. Neurosci. 22, 9698–9707. 10.1523/jneurosci.22-22-09698.2002 12427825 PMC6757844

[B21] Stocker M. Hirzel K. D’Hoedt D. Pedarzani P. (2004). Matching molecules to function: neuronal Ca^2+^-activated K^+^ channels and afterhyperpolarizations. Toxicon 43, 933–949. 10.1016/j.toxicon.2003.12.009 15208027

[B22] Stocker M. Krause M. Pedarzani P. (1999). An apamin-sensitive Ca^2+^-activated K^+^ current in hippocampal pyramidal neurons. Proc. Natl. Acad. Sci. U S A 96, 4662–4667. 10.1073/pnas.96.8.4662 10200319 PMC16389

[B23] Stocker M. Pedarzani P. (2000). Differential distributions of three Ca^2+^-activated K^+^ channel subunits, SK1, Sk2, and SK3, in the adult rat central nervous system. Mol. Cell. Neurosci. 15, 476–493. 10.1006/mcne.2000.0842 10833304

[B24] Strassmaier T. Bond C. T. Sailer C. A. Knaus H. G. Maylie J. Adelman J. P. (2005). A novel isoform of SK2 assembles with other SK subunits in mouse brain. J. Biol. Chem. 280, 21231–21236. 10.1074/jbc.m413125200 15797870

[B25] Vergara C. Latorre R. Marrion N. V. Adelman J. P. (1998). Calcium-activated potassium channels. Curr. Opin. Neurobiol. 8, 321–329. 10.1016/S0959-4388(98)80056-1 9687354

[B26] Xia X. M. Fakler B. Rivard A. Wayman G. Johnson-Pais T. Keen J. E.. (1998). Mechanism of calcium gating in small-conductance calcium-activated potassium channels. Nature 395, 503–507. 10.1038/26758 9774106

