# Supplementary material for: rSK1 in Rat Neurons: A Controller of Membrane rSK2?
Source: Front Neural Circuits. 2019 Apr 3;13:21. doi: 10.3389/fncir.2019.00021 (PMC6456674; doi:10.3389/fncir.2019.00021)
Supplement: Supplementary file 1 [file Data_Sheet_1.PDF]

Table 1. Passive and active membrane properties of infected neurons @ DIV15-17

	SCRAM (n = 15) (mean ± SEM)	rSK1-KD (n = 27) (mean ± SEM)	p value
Resting membrane potential (mV)	-55.0 ± 2.1	-50 ± 1.5	p = 0.07
Input resistance (MΩ)	320.3 ± 36.5	474.3 ± 56.3	p = 0.1
Membrane time constant (ms)	0.9 ± 0.1	0.8 ± 0.1	p = 0.7
Membrane capacitance (pF)	3.6 ± 0.7	3.0 ± 0.7	p = 0.7
